# Risk Factors for *Salmonella* Infection in Children under Five Years: A Hospital-Based Study in Kilimanjaro Region, Tanzania

**DOI:** 10.3390/pathogens13090798

**Published:** 2024-09-14

**Authors:** Ephrasia A. Hugho, Blandina T. Mmbaga, Abdul-Hamid S. Lukambagire, Grace D. Kinabo, Kate M. Thomas, Happiness H. Kumburu, Tine Hald

**Affiliations:** 1 Kilimanjaro Clinical Research Institute, Moshi 25102, Tanzanialukhamid@gmail.com (A.-H.S.L.);; 2 Faculty of Medicine, Kilimanjaro Christian Medical University College, Moshi 25102, Tanzania; 3 Department of Paediatrics, Kilimanjaro Christian Medical Centre, Moshi 25102, Tanzania; 4 EcoHealth Alliance, 520 Eighth Avenue, New York, NY 10018, USA; 5 New Zealand Food Safety, Ministry for Primary Industries, Wellington 6011, New Zealand; 6 Research Group for Genomic Epidemiology, Technical University of Denmark, 2800 Lyngby, Denmark

**Keywords:** salmonellosis, diarrhea, children, Tanzania

## Abstract

*Salmonella* is among the causative agents for diarrhea worldwide, but its risk factors in Tanzanian children are poorly understood. A hospital-based cross-sectional study was conducted in Moshi, Kilimanjaro region, from July 2020 to November 2022 among children under five admitted with diarrhea. A questionnaire was administered to all parents/caretakers of the enrolled children. Logistic regression was utilized to analyze the risk factors, with significance at *p* < 0.05. A total of 306 children were enrolled in the study. The median age was 13.8 months (IQR 8.4–21.8). The majority (58.5%) were males, and 59.5% were from rural areas. *Salmonella* was identified in eight (2.6%) stool samples, with a higher prevalence in urban than rural areas (4.8% vs. 1.1%; *p*-value = 0.044). The significant risk factors associated with *Salmonella* infection among the children included consuming raw milk (adjusted OR = 30.19; 95% CI: 3.94–231.46), using infant formula (adjusted OR = 15.78; 95% CI: 2.98–83.56), undisclosed household income (adjusted OR = 9.98; 95% CI: 2.46–40.12), purchasing eggs direct from the farms (adjusted OR = 7.58; 95%CI: 1.31–43.96), and contact with chickens (adjusted OR = 6.49; 95%CI: 1.25–33.59). These findings highlight the need for targeted interventions to improve food safety, hygiene practices, and socioeconomic conditions.

## 1. Introduction

Diarrhea remains a significant cause of child mortality and was responsible for approximately 9% of all deaths among children under the age of 5 worldwide in 2019. This equates to more than 1200 young children succumbing to the condition daily or approximately 444,000 children annually [1]. Diarrhea can have many causes, including infection with *Salmonella* species [2]. While many cases of salmonellosis are mild, there are occasions when it can pose a life-threatening risk. The gravity of the illness is conditional on both host-specific factors and the specific serotype of *Salmonella* involved [3]. It is estimated that approximately 93.8 million cases of gastroenteritis attributed to nontyphoidal *Salmonella* species occur annually on a global scale, resulting in 155,000 fatalities [4].

Salmonellosis in humans is typically acquired by consuming food of animal origin, particularly items such as eggs, meat, poultry, and milk, which may be contaminated [5,6]. However, there have been cases where other foods, such as green vegetables exposed to animal waste, have been linked to its transmission [7]. It can also be transmitted from person to person through the fecal–oral route. Additionally, human cases may arise when individuals come into contact with infected animals, including pets [7,8,9]. Salmonellosis typically manifests as a sudden onset of symptoms, typically fever, abdominal pain, diarrhea, nausea, and occasional vomiting. Infants and children under five are among the high-risk groups [10].

In developed countries, *Salmonella* infections are primarily linked to animal contact and food products, often leading to outbreaks, with children under five bearing a higher burden. In the United States, young children are disproportionately affected by these infections [11]. In Canada, the mean annual number of nontyphoidal *Salmonella* cases exceeds 150,000, including 1565 hospitalizations and 29 deaths when accounting for underreporting [12]. In Europe, 4.9% of acute inflammatory diarrhea cases in children in Georgia are due to *Salmonella* [13]. Similarly, in Uruguay, *Salmonella* causes 5% of acute diarrhea cases in children [14].

In developing countries, diarrhea cases caused by *Salmonella* vary between geographical locations. In Malawi, *Salmonella* species were identified in 2.3% of diarrhea cases and 0.4% in the control group [15], while 10.3% to 12.7% of cases were due to *Salmonella* Typhimurium in Iraq [16,17]. In Sudan, *Salmonella* Typhi and *Salmonella* Paratyphi were isolated from children with diarrhea at a prevalence of 4% [18]. In Niger, *Salmonella* was responsible for 9.2% of diarrhea cases, with serotypes Typhimurium, Enteritidis, and Corvalis being the most common serotypes [19].

In Tanzania, diarrheal disease continues to be a significant health concern for young children. According to the 2022 Tanzania Demographic and Health Survey and Malaria Indicator Survey (TDHS-MIS) report, the overall national prevalence of diarrhea among under five years children was 9% [20]. The TDHS-MIS report indicated that children between 6 and 23 months of age are particularly vulnerable, with a higher prevalence of diarrhea (15–19%). As children reach the age of 2, the prevalence of diarrhea gradually decreases, reaching as low as 3% among those aged 48–59 months [20]. To mitigate diarrhea incidences, simple interventions, including access to safe water, adequate sanitation, hand washing, and routine rotavirus vaccination, have been recommended. However, the contribution of specific pathogens to diarrhea incidence, particularly due to/caused by *Salmonella,* is likely underreported. Few studies have been conducted to investigate *Salmonella* as an etiological agent for diarrhea in children in Tanzania. These include a study conducted in Morogoro among children with diarrhea, which showed that *Salmonella* species were detected in low percentages (1.4%) compared to other enteropathogens, such as *Shigella* and diarrheagenic *E. coli* [21]. Similarly, another study in the Dar es Salaam region found a prevalence of *Salmonella* infection of 2.5 [22]. Compounding the scarcity of available evidence to confirm *Salmonella* as one of the causative agents for diarrhea in Tanzanian children, few studies have reported any environmental factors that could contribute to the transmission of *Salmonella* species to children. This study investigated the risk factors for *Salmonella* infection in children with diarrhea in the Kilimanjaro region, building upon a previous study that used the One Health approach to investigate *Salmonella* from children and the surrounding household environment.

## 2. Materials and Methods

### 2.1. Study Design and Setting

This study employed a cross-sectional design using data collected from a hospital-based survey and microbiological culture. The data were originally collected as a part of foodborne disease epidemiology, surveillance, and control in African low- and middle-income countries (FOCAL project) from July 2020 to November 2022 in Moshi, Kilimanjaro region.

### 2.2. Study Population

The study population was comprised of children under five years with diarrhea. The inclusion criteria were admission with diarrhea at health facilities in Moshi and those whose parents or caretakers provided consent.

### 2.3. Data Collection Tool and Method

A structured questionnaire was developed to assess sources of diarrheal disease in under five children as part of the FOCAL project. The questions were selected based on a review of the literature on diarrhea disease in young children, consultations with public health experts, and guidelines from the WHO to ensure that they covered the most relevant environmental, behavioral, and socioeconomic factors associated with diarrheal diseases. The questionnaire included closed-ended questions to collect standardized data.

Pre-testing of the tool was conducted with a small sample of five volunteers, who were representative of the target population. These volunteers included parents or caregivers of under five children to assess the clarity and consistency of the responses. The feedback from this process was considered in refining the questionnaire. Based on the volunteers’ suggestions, certain questions were rephrased for better clarity, additional response options were added, and irrelevant or redundant questions were removed.

The final version of the questionnaire was administered in the local language to all parents or caretakers of the enrolled children. The questionnaire covered general demographic information; disease-related information (including signs and symptoms); food eaten in the household (including food purchase habits); water supply (including water sources for drinking, food preparation, washing, bathing, and swimming); the toilet system; contact with animals; and occupation. The responses were recorded in an electronic database, EpiInfo version 7.2.5 (EpiInfo^TM^, Centers for Disease Control and Prevention, Atlanta, Georgia).

### 2.4. Stool Culture

Stool samples were collected from the admitted children and cultured to isolate *Salmonella* species. The culture tests were performed at Kilimanjaro Clinical Research Institute laboratory using standard microbiological techniques. The results of these culture tests were reported in the initial study [23].

### 2.5. Data Analysis

STATA version 15 (STATA Corp, College Station, TX, USA) was used for data analysis. Categorical variables were summarized by frequency and proportion, while the numeric variables were summarized by measures of central tendency and their corresponding measures of dispersion, based on the nature of their distributions. Variable re-categorization was performed to ensure adequate distribution of the observations for the regression analysis. Logistic regression was used to estimate the factors associated with *Salmonella* infection in both the bivariate and multivariable models. Stepwise logistic regression using backward selection with a probability of 0.2 was used to find the best model. The best-fitted model was chosen based on Akaike Information Criterion (AIC). Variance inflation factor (VIF) was used to check for multicollinearity between the variables retained in the stepwise model. Factors with a *p*-value < 0.05 were considered statistically significant.

## 3. Results

### 3.1. Sociodemographic Characteristics of the Study Participants

A total of 306 children were enrolled with the median age of 13.8 months (IQR 8.4–21.8). Fifty-eight percent (*n* = 179/306) were males, forty-three point one percent were aged less than one year, and fifty-nine point five percent of them were residing in rural areas. The majority had vomiting and fever (Table 1).

### 3.2. Prevalence of Salmonella spp. Among Children with Diarrhea

Of 306 stool samples tested for the presence or absence of *Salmonella* species, 8 samples (2.6%) were *Salmonella* culture-positive. The infection rate was higher among children living in urban areas (4.8% [6/124] vs. 1.1% [2/182]; *p* = 0.044), despite being equally distributed among children’s sex. Half of the *Salmonella*-positive cases (4/8) were detected among children aged less than 1 year. Vomiting was reported in all *Salmonella*-positive cases, fever in six (75.0%) cases, and four (50%) among children whose household income was not disclosed (Table 1).

### 3.3. Salmonella Prevalence in Relation to Sources of Water, Household Living Conditions, and Toilet Systems

Over 70% of the respondents had access to piped water in their homes as their primary source of water for drinking, food preparation, washing, and bathing. Other sources reported by at least 5% included private wells or pumps (protected) and public/communal wells or standpipes. More than 75% of *Salmonella*-positive cases were from children whose households had access to piped water, used for drinking, food preparation, and washing household utensils. The majority, 67.5% (*n* = 206), were aware of the need to treat drinking water, and the common treatment method was the addition of disinfectants such as chlorine. Of those who reported swimming, the main source was river water 73% (*n* = 9/12). However, there were no *Salmonella*-positive cases found in this group (Table 2).

Most of the households had more than one fuel option for cooking. The overall fuel sources used in these households were gas, charcoal, and firewood. Despite that seven out of eight *Salmonella* culture-positive cases were from children whose households used cooking gas as the main fuel, this association was not significant. Children from households reporting the consumption of raw milk and infant formula in the past two weeks were found to have a significant prevalence of *Salmonella* (22.2% and 15.0%, respectively; Fisher’s exact *p*-value < 0.05). Moreover, most of the houses had improved toilet systems, with the majority (71.6%, *n* = 219) having flush or pour toilets with septic tanks, followed by flush or pour toilets connected to a sewer pipe (16.7%, *n* = 51). Despite the fact that five out of eight (62.5%) of the *Salmonella*-positive cases were from children living in households with flush or pour toilets connected to a sewer pipe and those with more than three rooms, the difference in distribution was not significant (Table 3).

### 3.4. Domestic Animal Ownership and Children’s Interaction with Animals

Fifty-one percent of the enrolled children were living in the households with domestic animals in their surrounding environment (*n* = 159). The majority, 89.3% (*n* = 142), had chickens, followed by cattle 33.3% (*n* = 53) and goats 32.7% (*n* = 52). Ninety-four (94.4%) households reported keeping domestic animals located around the house areas. Children’s contact with cattle, goats, sheep, chickens, ducks, dogs, cats, and rabbits was reported. However, contact with chickens was most common (44.4%), followed by goats, cattle, and ducks. The contact frequency differed between animal species, but the majority reported daily and one to three times per week. Among the 63 children who were reported to have had contact with chickens, 2 (3.2%) were infected with *Salmonella*. One child (14.3%) among the seven who were reported to have had contact with ducks was infected with *Salmonella* (Table 4).

### 3.5. Factors Associated with Salmonella Infection

Bivariate analysis of the association between the selected risk factors and *Salmonella* infection among all the children revealed that children from households with an undisclosed monthly income had a significantly higher risk of *Salmonella* infection compared to those from households with disclosed incomes of less than 200,000 TZS (OR = 16.58; 95% CI: 2.88–95.37). Consumption of raw milk was associated with more than 13 times the odds of *Salmonella* infection compared to those who did not consume raw milk (OR = 13.86; 95% CI: 2.36–81.36). Additionally, children using infant formula had 12 times the odds of *Salmonella* infection compared to those who did not use infant formula (OR = 12.58; 95% CI: 2.34–67.19). Other factors, such as caretaker’s occupation and household living conditions, did not show any significant association with *Salmonella* infection.

In the multivariable analysis, children from households that reported consuming raw milk in the weeks prior to the study had significantly higher odds of *Salmonella* infection compared to those who did not consume raw milk (adjusted OR = 30.19; 95% CI: 3.94–231.46). Additionally, children using infant formula (adjusted OR = 15.78; 95% CI: 2.98–83.56) and those from households with an unknown income status (adjusted OR = 9.98; 95% CI: 2.46–40.12) had higher odds of testing positive for *Salmonella* culture compared to their counterparts. Purchasing eggs from farms was associated with seven times the odds of *Salmonella* infection as compared to those who did not (adjusted OR = 7.58; 95% CI: 1.31–43.96). Moreover, children reported to have had contact with chickens had higher odds of *Salmonella* infection compared to those reported to have had no contact with chickens (adjusted OR = 6.49; 95% CI: 1.25–33.59) (Table 5).

## 4. Discussion

This study reports the risk factors for salmonellosis among children under five admitted with diarrhea in rural and urban districts of the Kilimanjaro region. The factors significantly associated with *Salmonella* infection were monthly income, consumption of raw milk, infant formula, purchasing eggs from farms, and contact with chickens.

The prevalence of *Salmonella* in children under five admitted with diarrhea in the study area was 2.6%, higher than the prevalence reported previously in the Morogoro region (1.4%) and Dar es Salaam region (2.5%) [21,22]. However, the reported prevalence in our study area was lower than the prevalence reported as 10.3% in Iraq, 2.6% in Malawi, 8.9% in Niger, and 4.0% in Sudan [15,17,18,19]. A high prevalence of *Salmonella* has also been reported among children with diarrhea in China [24], Ethiopia [25], and Kenya [26]. The differences in these findings may be explained by different study designs and variations in sample sizes. The lower prevalence of *Salmonella* found in diarrhea cases in this study likely indicates less exposure to *Salmonella.* Our study found that 50% of *Salmonella*-positive cases were from children aged less than one year, despite showing no significant difference by age. This can be attributed to the higher proportion of children under one year (43%) enrolled in the study, which is influenced by caretakers’ habits of seeking medical assistance for their young children. More diarrhea cases during this period also coincide with the transition from breastfeeding, as maternal antibodies wane and as children become more mobile, increasing their exposure to potentially contaminated food, water, and surroundings. Despite our study finding a non-significant association between child age and risk of *Salmonella* infection, a higher *Salmonella* prevalence among under five children compared to adults has been reported elsewhere [27,28].

Household income, consumption of raw milk, and infant formula demonstrated a significant relationship with *Salmonella* infection. This study found that children from households with an undisclosed monthly income had a significantly higher risk of *Salmonella* infection than those with a disclosed income of less than 200,000 TZS. This finding suggests that an undisclosed income might indicate economic instability or reluctance to disclose financial details, which could be associated with poor living conditions, lack of access to clean water, inadequate sanitation, and limited healthcare access. Several studies have shown a correlation between socioeconomic status and higher rates of infectious diseases, including *Salmonella*. For instance, a study in Canada reported that low-income households had a higher prevalence of *Salmonella* and other enteric infections due to poor hygiene and limited access to clean water and sanitation facilities [29]. However, other studies reported an increased infection rate associated with high income, which is likely to affect the dietary habits [30].

The consumption of raw milk was associated with more than 30 times the odds of *Salmonella* infection compared to those who did not consume raw milk. Raw milk can be a vehicle for various pathogens, including *Salmonella*, due to the absence of pasteurization, which kills harmful bacteria. Numerous studies have supported the association between raw milk consumption and *Salmonella* infection. A review article by Oliver and colleagues highlighted multiple outbreaks of *Salmonella* linked to raw milk consumption, highlighting the significant health risks associated with this practice [31]. Another study in the United States reported outbreak cases related to *Salmonella* spp. and other foodborne pathogens such as *Campylobacter, E. coli, Listeria,* and *Brucella* [32]. With this situation, the consumption of raw milk has been strongly advised against due to its potential to harbor harmful bacteria, including *Salmonella, E. coli*, and *Listeria* [33]. Additionally, children using infant formula had 15 times higher odds of *Salmonella* infection compared to those who did not use infant formula. This could be linked to the preparation and storage conditions of infant formula, such as inadequate sterilization of feeding bottles and improper formula preparation techniques, which can be prone to contamination if not handled properly. Similar findings were reported in Israel and the United States, documenting an increased risk of *Salmonella* infection for children using milk formula [34,35]. Studies have also documented cases of *Salmonella* outbreaks associated with contaminated infant formula in France and Spain [36,37].

Purchasing eggs directly from farms was associated with higher odds of *Salmonella* infection than purchasing them elsewhere. Eggs purchased from farms are often fresher than those from shops or supermarkets. Fresh eggs may have a lower risk of contamination if handled properly, since *Salmonella* can multiply over time in improperly stored eggs [38,39]. However, unlike larger farms producing commercial eggs that normally undergo sterilization to kill *Salmonella,* eggs from small farmers are less likely to be treated to kill *Salmonella* before selling [40]. This ultimately increases the risk of *Salmonella,* especially if the eggs are consumed raw or undercooked. Further, this study was conducted in areas where there are small farms, hence practices such as regular testing for *Salmonella,* proper storage, and handling might be less stringent, increasing the risk of contamination. Additionally, exposure to infected flocks may occur if the farm does not monitor *Salmonella* regularly, leading to the production of contaminated eggs from infected hens [41]. Therefore, small farmers should follow good farming practices, handling, and storage of eggs, while consumers must ensure the thorough cooking of eggs and proper hygiene when handling, including washing hands, utensils, and surfaces that come into contact with raw eggs.

Moreover, contact with chickens was significantly associated with higher odds of *Salmonella* infection. Households with chickens have been reported as a significant risk factor for infection with other *Salmonella* serotypes (*Salmonella* Typhi) in Kenya compared to those without [42]. Another study in Kenya found that, for each additional sheep kept in the house, the risk of diarrhea increased 1.2-fold. The presence of rodents and chickens interacting with children increased the likelihood of diarrhea 7.5- and 3.8-fold, respectively [26]. Another study in Uganda reported an 83% increase in the two-week reported prevalence of diarrhea among children less than five years from households with an above median of five chickens as compared to those with fewer chickens [43]. A study conducted in Michigan reported a 24-fold increase in the risk of *Salmonella* infection among children who had contact with cats [44]. Domestic animals/pets are reservoirs of many zoonotic pathogens, as evidenced by the isolation of some pathogens from animal excreta [26,45,46]. Generally, contact with animals is an important risk factor for *Salmonella* infection, given its zoonotic nature and wide range of hosts [47,48]. Therefore, it is important to restrict children’s movements to areas close to domestic animals and/or to minimize the risk of acquiring zoonotic pathogens.

It is essential to point out that this study was carried out during the COVID-19 pandemic. Hygiene practices implemented in the communities, especially hand-washing hygiene, were the key intervention for preventing diarrhea. Variations in salmonellosis before and after COVID-19 have been reported in some places. For instance, there has been a sharp increase in nontyphoidal *Salmonella*-positive cases in China of 66.5%, from 4.90% to 8.16%, before and after COVID-19, respectively [49]. The increase was attributed to higher nontyphoidal *Salmonella* fevers before COVID-19 as compared to other enteric pathogens, which could have prompted high medical care-seeking behavior and increased the *Salmonella*-positive rate. In contrast, a reduction in *Salmonella* incidence from 2.1 to 1.4 per 100,000 people has been reported in Israel [50], and a decrease of between 37% and 55% was reported in the Netherlands after the implementation of COVID-19 pandemic control measures [51]. However, the impact of the COVID-19 pandemic on *Salmonella* infections in Tanzania has not yet been reported. Our findings might have been affected by improved hygiene as a result of COVID-19 control measures.

## 5. Limitations of the Study

This study was conducted among children admitted to healthcare facilities with diarrhea. Hence, a possible limitation of our findings may include the lack of a control group (children without diarrhea) from the same communities, as well as outpatient children with diarrhea, which might underestimate the prevalence of *Salmonella* and the potential effects of the exposures assessed. Sample size would also be a limiting factor, as other studies that enrolled a higher number of participants were able to obtain significant findings in some of the risk factors. The presence of undisclosed income data among households may have affected the accuracy of our findings. Households with undisclosed income could be different from those that reported their income, potentially due to socioeconomic factors or a reluctance to disclose financial information. This non-disclosure can obscure the true relationship between income levels and the risk of *Salmonella* infection, leading to an underestimation or overestimation of the association. Lastly, the study was conducted after the COVID-19 pandemic; hence, the majority of people had an increased awareness of good hygiene.

## 6. Conclusions

This study contributes to the data on *Salmonella* gastroenteritis in Tanzania and identifies the risk factors associated with *Salmonella* infection among children under five in the Kilimanjaro region. The prevalence of *Salmonella* was higher in urban areas than rural areas, with raw milk consumption, infant formula use, household income, purchasing eggs direct from the farms, and contact with chickens being the key determinants of infection risk. These findings highlight the need for targeted interventions to improve food safety, hygiene practices, and socioeconomic conditions.

## Figures and Tables

**Table 1:** Demographics, clinical characteristics, and prevalence of *Salmonella* spp. among the study participants (N = 306).

Characteristic	Total [*n* (%)]	*Salmonella* −ve [*n* (%)]	*Salmonella* +ve [*n* (%)]
**Child sex**			
Female	127 (41.5)	123 (96.9)	4 (3.1)
Male	179 (58.5)	175 (97.8)	4 (2.2)
**Child age (months)**			
**Median (IQR)**	13.8 (8.3–21.8)		
**0** to 11	132 (43.1)	128 (97.0)	4 (3.0)
12 to 23	105 (34.3)	103 (98.1)	2 (1.9)
24 to 59	69 (22.5)	67 (97.1)	2 (2.9)
**Clinical signs/symptoms**			
Vomiting	268 (87.6)	260 (97.0)	8 (3.0)
Fever	190 (62.1)	184 (96.8)	6 (3.2)
Loss of appetite	110 (35.9)	109 (99.1)	1 (0.9)
Coughing	118 (38.6)	116 (98.3)	2 (1.7)
Running nose	109 (35.6)	109 (100.0)	0
Fatigue	47 (15.4)	45 (95.7)	2 (4.3)
Abdominal pain	22 (7.2)	21 (95.5)	1 (4.5)
Nausea	11 (3.6)	11 (100.0)	0
Flatulence	21 (6.9)	20 (95.2)	1 (4.8)
Headache	1 (0.3)	1 (100.0)	0
Jaundice	1 (0.3)	1 (100.0)	0
Dizziness	1 (0.3)	1 (100.0)	0
**Residence**			
Rural	182 (59.5)	180 (98.9)	2 (1.1)
Urban	124 (40.5)	118 (95.2)	6 (4.8)
**Caretakers’ employment status**			
Housewife	101 (33.0)	98 (97.0)	3 (3.0)
Employed/self-employed	205 (77.0)	200 (97.6)	5 (2.4)
**Household income (TZS)**			
<200,000	201 (65.7)	199 (99.0)	2 (1.0)
≥200,000	77 (25.2)	75 (97.4)	2 (2.6)
Not disclosed	28 (9.1)	24 (85.7)	4 (14.3)

**Table 2:** Water sources used by the household for domestic purposes and *Salmonella* spp. prevalence (N = 306).

Water Source	* Salmonella * −ve *n* (%)	* Salmonella * +ve *n* (%)	Total *n* (%)
**Drinking water**			
Piped water into home	250 (97.3)	7 (2.7)	257 (84.0)
Public tap/standpipe	32 (100.0)	0	32 (10.5)
Protected well or spring	7 (100.0)	0	7 (2.3)
Unprotected well or spring	0	1 (100.0)	1 (0.3)
River	2 (100.0)	0	2 (0.7)
Other *	7 (100.0)	0	7 (2.3)
**Water for food preparation**			
Piped water into home	232 (97.1)	7 (2.9)	239 (78.1)
Public tap/standpipe	21 (100.0)	0	21 (6.9)
Protected well or spring	31 (100.0)	0	31 (10.1)
Unprotected well or spring	2 (66.7)	1 (33.3)	3 (1.0)
River	6 (100.0)	0	6 (2.0)
Other *	6 (100.0)	0	6 (2.0)
**Wash water**			
Piped water into home	216 (97.3)	6 (2.7)	222 (72.5)
Public tap/standpipe	22 (100.0)	0	22 (7.2)
Protected well or spring	42 (97.7)	1 (2.3)	43 (14.1)
Unprotected well or spring	2 (66.7)	1 (33.3)	3 (1)
River	8 (100.0)	0	8 (2.6)
Other *	8 (100.0)	0	8 (2.6)
**Bath water**			
Piped water into home	212 (97.2)	6 (2.8)	218 (71.2)
Public tap/standpipe	23 (100.0)	0	23 (7.5)
Protected well or spring	43 (97.7)	1 (2.3)	44 (14.4)
Unprotected well or spring	3 (75.0)	1 (25.0)	4 (1.3)
River	8 (100.0)	0	8 (2.6)
Other *	9 (100.0)	0	9 (2.9)
**Swimming**			
River	9 (100.0)	0	9 (81.8)0
Other	2 (100.0)	0	2 (18.2)0

* Water from creek, cart, or wheelbarrow with small tank or drum, pond, or dam.

**Table 3:** *Salmonella* prevalence in relation to household living conditions, cooking fuels, and the toilet system.

Characteristics	*Salmonella* −ve *n* (%)	*Salmonella* +ve *n* (%)	Total *n* (%)
**Place of food Purchase**			
**Meat and Poultry**			
Butcher meat	265 (97.4)	7 (2.6)	272 (88.9)
Market meat	9 (90.0)	1(10.0)	10 (3.3)
Market poultry	37 (92.5)	3 (7.5)	40 (13.1)
Self-sufficient poultry	107 (98.2)	2 (1.8)	109 (35.6)
**Eggs**			
Direct from farm	7 (87.5)	1 (12.5)	8 (2.6)
Self-sufficient eggs	106 (98.1)	2 (1.9)	108 (35.3)
Other	44 (95.6)	2 (4.4)	46 (15.0)
**Fish**			
Market	140 (96.6)	5 (3.4)	145 (47.4)
Other	83 (97.7)	2 (2.3)	85 (27.8)
**Food eaten**			
Raw milk	7 (77.8)	2 (22.2)	9 (2.9)
Beef	181 (97.3)	5 (2.7)	186 (60.8)
Chicken	43 (97.7)	1 (2.3)	44 (14.4)
Fish	145 (97.3)	4 (2.7)	149 (48.7)
**Food for infants (*n* = 237)**			
Infant formula	17 (85.0)	3 (15.0)	20 (6.5)
Fruit puree	5 (83.3)	1 (16.7)	6 (2.5)
Fermented cereals	161 (98.2)	3 (1.8)	164 (69.2)
Fruit or vegetable juice	21 (95.4)	1 (4.56	22 (9.3)
Milk breastfeeding	151 (97.4)	4 (2.6)	155 (65.4)
**Rooms per household**			
1	97 (99.0)	1 (1.0)	98 (32.0)
2	103 (98.1)	2 (1.9)	105 (34.3)
3 or more	98 (95.1)	5 (4.9)	103 (33.7)
**Persons per household**			
2 to 3	111 (98.2)	2 (1.8)	113 (36.9)
4 to 5	118 (97.5)	3 (2.5)	121 (39.5)
6 or more	69 (95.8)	3 (4.2)	72 (23.5)
**Persons per room**			
2 to 3	211 (97.7)	5 (2.3)	216 (70.6)
3 or more	87 (96.7)	3 (3.3)	90 (29.4)
**Cooking fuel type**			
Cooking gas	234 (97.1)	7 (2.9)	241 (78.8)
Charcoal	187 (97.4)	5 (2.6)	192 (62.7)
Firewood	130 (98.5)	2 (1.5)	132 (43.1)
Kerosene	36 (97.3)	1 (2.7)	37 (12.1)
Electricity	18 (97.4)	1 (5.3)	19 (6.2)
**Toilet system**			
Flush toilet connected to sewer pipe	214 (97.7)	5 (2.3)	219 (71.6)
Flush toilet with septic tank	50 (98.0)	1 (2.0)	51 (16.7)
Pit latrine with covering slab	34 (94.4)	2 (5.6)	36 (11.8)

**Table 4:** Interactions with animals among children with diarrhea that reported household animal ownership; Moshi, 2020–2022 (*n* = 159).

Animal Species	Number of Children Living in Households with Animals Present (%)	Number of Children Reported to Have Contact with Household Animals (%)	Household Animal Contact Frequency			*Salmonella* +ve
			Daily	4–6 times/week	1–3 times/week	
Cattle	53 (33.3)	7 (13.2)	2 (28.6)	1 (14.3)	4 (57.1)	-
Goats	52 (32.7)	11 (21.1)	4 (36.4)	2 (18.2)	5 (45.4)	-
Sheep	13 (8.2)	2 (15.4)	1 (50.0)	-	1 (50.0)	-
Pigs	14 (8.8)	1 (7.1)	-	-	1 (100.0)	-
Chicken	142 (89.3)	63 (44.4)	25 (39.7)	19 (30.2)	19 (30.2)	2 (3.2)
Ducks	22 (13.8)	7 (31.8)	1 (12.2)	4 (50.0)	3 (37.5)	1 (14.3)
Dogs	6 (3.8)	1 (16.7)	1 (100.0)	-	-	-
Cats	4 (2.5)	2 (50.0)	1 (50.0)	-	1 (50.0)	-
Donkeys	1 (0.6)	-	-	-	-	-
Rabbit	4 (2.5)	2 (50.0)	1 (50.0)	-	1 (50.0)	-

**Table 5:** Bivariate and multivariable analyses of the factors associated with *Salmonella* infection (N = 306).

Variable	*Salmonella* +ve *n* (%)	Unadjusted OR (95% CI)	*p*-Value	Adjusted OR (95% CI)	*p*-Value
**Child age (Months)**					
0–11	4 (3.0)	Ref			
12–23	2 (1.9)	0.62 (0.11–3.47)	0.588		
24–59	2 (2.9)	0.9.6 (0.17–5.36)	0.959		
**Child sex**					
Female	4 (3.1)	Ref			
Male	4 (2.2)	0.70 (0.17–2.87)	0.623		
**Caretakers’ employment status**					
Housewife	3 (3.0)	Ref			
Employed/self-employed	5 (2.4)	0.82 (0.19–3.49)	0.785		
**Household income (TZS)**					
<200,000	2 (1.0)	Ref			
≥200,000	2 (2.6)	2.68 (0.38–19.28)	0.334		
Not disclosed	4 (14.3)	16.58 (2.88–95.37)	0.002	9.98 (2.46–40.12)	0.001
**Residence**					
Rural	2 (1.1)	Ref			
Urban	6 (4.8)	4.58 (0.90–23.12)	0.066	3.87 (0.76–19.59)	0.102
**Place of food Purchase**					
**Meat and Poultry**					
Butcher meat	7 (2.6)	0.87 (0.10–7.33)	0.899		
Market meat	1(10.0)	4.58 (0.51–41.46)	0.175		
Market poultry	3 (7.5)	4.23 (0.96–18.49)	0.055		
Self-sufficient poultry	2 (1.8)	0.60 (0.12–3.01)	0.530		
**Eggs**					
Direct from farm	1 (12.5)	5.94 (0.64–55.17)	0.117	7.58 (1.31–43.96)	0.024
Self-sufficient eggs	2 (1.9)	0.60 (0.12–3.05)	0.542		
Other	2 (4.4)	1.92 (0.38–9.87)	0.433		
**Fish**					
Market	5 (3.4)	1.88 (0.44–8.03)	0.394		
Other	2 (2.3)	0.86 (0.17–4.38)	0.859		
**Food eaten past two weeks**					
Raw milk	2 (22.2)	13.86 (2.36–81.36)	0.004	30.19 (3.94–231.46)	0.001
Beef	5 (2.7)	1.08 (0.25–4.60)	0.920		
Chicken	1 (2.3)	0.85 (0.10–7.08)	0.878		
Fish	4 (2.7)	1.06 (0.26–4.31)	0.940		
Infant formula	3 (15.0)	9.92 (2.17–45.13)	0.003	15.78 (2.98–83.56)	0.001
Fruit puree	1 (16.7)	8.37 (0.86–81.67)	0.068		
Fermented cereals	3 (1.8)	0.49 (0.12–2.12)	0.345		
Fruit or vegetable juice	1 (4.3)	1.79 (0.21–15.28)	0.536		
Milk breastfeeding	4 (2.6)	0.96 (0.25–3.92)	0.947		
**Animals in the household**					
Pigs	1 (7.1)	3.13 (0.36–27.47)	0.303		
Goats	1 (1.9)	0.69 (0.08–5.77)	0.733		
Chicken	2 (1.4)	0.38 (0.07–1.89)	0.237		
Ducks	1 (4.5)	1.88 (0.22–16.10)	0.563		
**Contact with animal**					
Chicken	2 (3.2)	1.32 (0.26–6.73)	0.091	6.49 (1.25–33.59)	0.026
Ducks	1 (14.3)	6.95 (0.73–65.93)	0.737		
**Household living conditions**					
**Toilet system**					
Flush toilet with septic tank	5 (2.2)	Ref			
Flush toilet connected to sewer	1 (2.0)	0.86 (0.09–7.52)	0.888		
Pit latrine	2 (5.5)	2.52 (0.47–13.53)	0.282		
**Number of rooms**					
1	1 (1.0)	Ref			
2	2 (1.9)	1.88 (0.17–21.19)	0.608		
3 or more	5 (4.8)	4.95 (0.56–43.29)	0.148	4.11 (0.99–17.00)	0.051
**Persons per household**					
2 to 3	2 (1.8)	Ref			
4 to 5	3 (2.5)	1.41 (0.23–8.63)	0.709		
6 or more	3 (4.2)	2.41 (0.39–14.85)	0.342		
**Persons per room**					
2–3	5 (2.3)	Ref			
4 or more	3 (3.3)	1.45 (0.34–6.24)	0.613		

OR—odds ratio; CI—confidence interval.

## Data Availability

The datasets used and/or analyzed during the current study are available from the corresponding author upon reasonable request.
